# Extensive Osteochondroma of the Talus Presenting As Syndesmotic Joint Extension and Posterior Inferior Tibiofibular Ligament Rupture: A Reportof a Rare Case and a Review of the Literature

**DOI:** 10.7759/cureus.55339

**Published:** 2024-03-01

**Authors:** Kamal Dureja, Pratheeksh P Suvarna, Amit K Sahu

**Affiliations:** 1 Department of Foot and Ankle Orthopedics, Max Smart Super Speciality Hospital, Saket, New Delhi, IND; 2 Department of Radiology, Max Smart Super Speciality Hospital, Saket, New Delhi, IND

**Keywords:** talus tumor, surgical fixation, pediatric, syndesmotic disruption, unusual site of osteochondroma

## Abstract

This case report describes a rare occurrence of talar osteochondroma extending into syndesmosis, causing disruption of the interosseous membrane and the posterior inferior tibiofibular ligament (PITFL). This type of presentation for a talar osteochondroma is the first of its kind reported in the literature based on current knowledge. A detailed preoperative radiological assessment was crucial in planning the surgical approach and preparing for syndesmotic stabilization during the excision. The patient underwent successful and complete excision of the osteochondroma, and the syndesmosis was stabilized using a cortical screw along with anatomical repair of the PITFL. Apart from delayed wound healing, the patient exhibited good functional outcomes in terms of gait and ankle range of motion at the six-month follow-up. This case serves as a valuable reference for similar presentations in the future, emphasizing the importance of thorough preoperative assessment and appropriate treatment planning.

## Introduction

Osteochondroma, referred to as osteochondromatous exostosis, osseocartilaginous exostosis, or simply exostosis, is defined by the World Health Organization as bony projections covered by cartilage that emerge on the outer surface of a bone. Despite being primarily composed of bone, their growth occurs within the cartilaginous segment [1]​.

The ongoing debate revolves around whether osteochondroma should be classified as a developmental disorder (pseudo-tumoral lesion) or as a neoplasm. 

There are two distinct clinical forms: solitary lesions (solitary osteochondromas) and multiple lesions (multiple osteochondromas). The solitary form accounts for 10% of all bone tumors, and within this category, it constitutes 35% (20-50%) of benign tumors [2]​.

Individuals diagnosed with osteochondroma are mostly found to have single lesions, which are typically identified during childhood or adolescence [1].​ 

## Case presentation

We were presented in the OPD with a seven-year-old male child complaining of pain and swelling around his right ankle joint while playing for three months. There was no history of trauma, fever, loss of weight or appetite.

**Figure 1:**
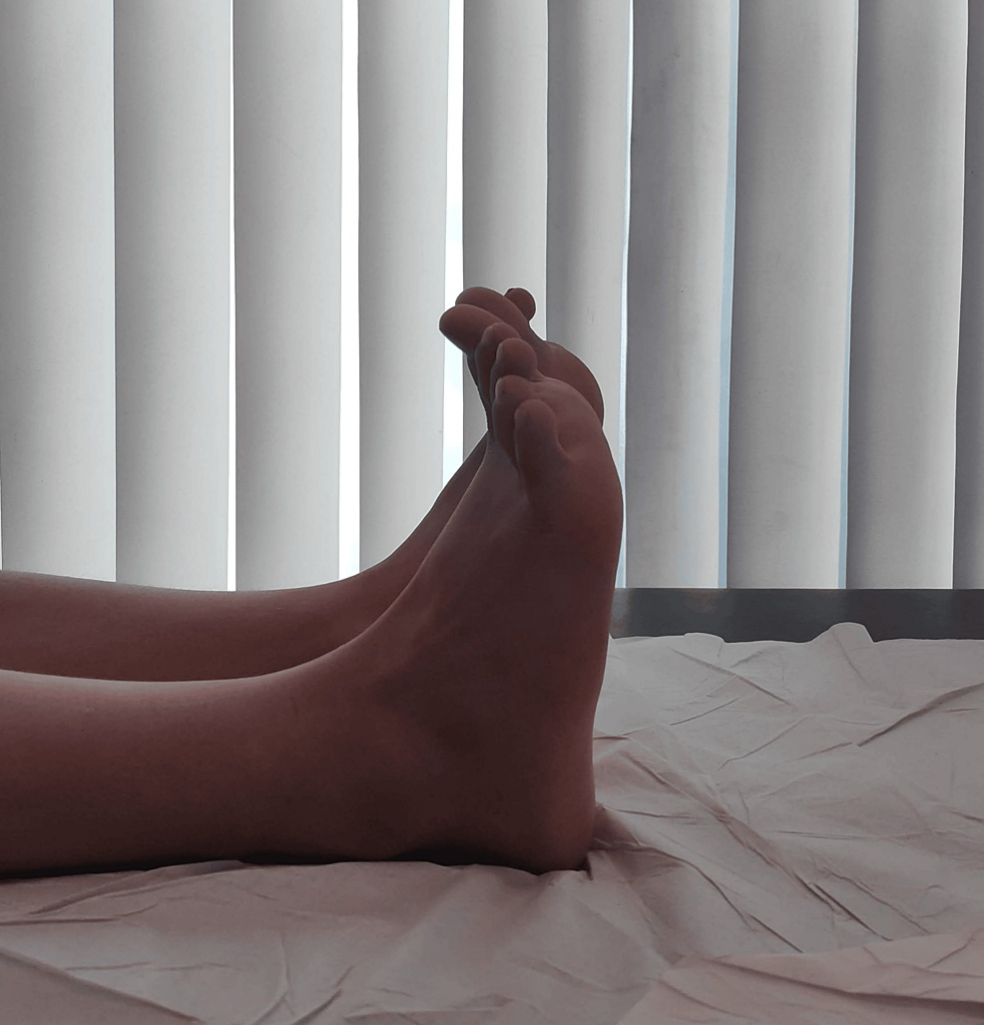
Preoperative photo of ankle demonstrating dorsiflexion of 0 degrees

**Figure 2:**
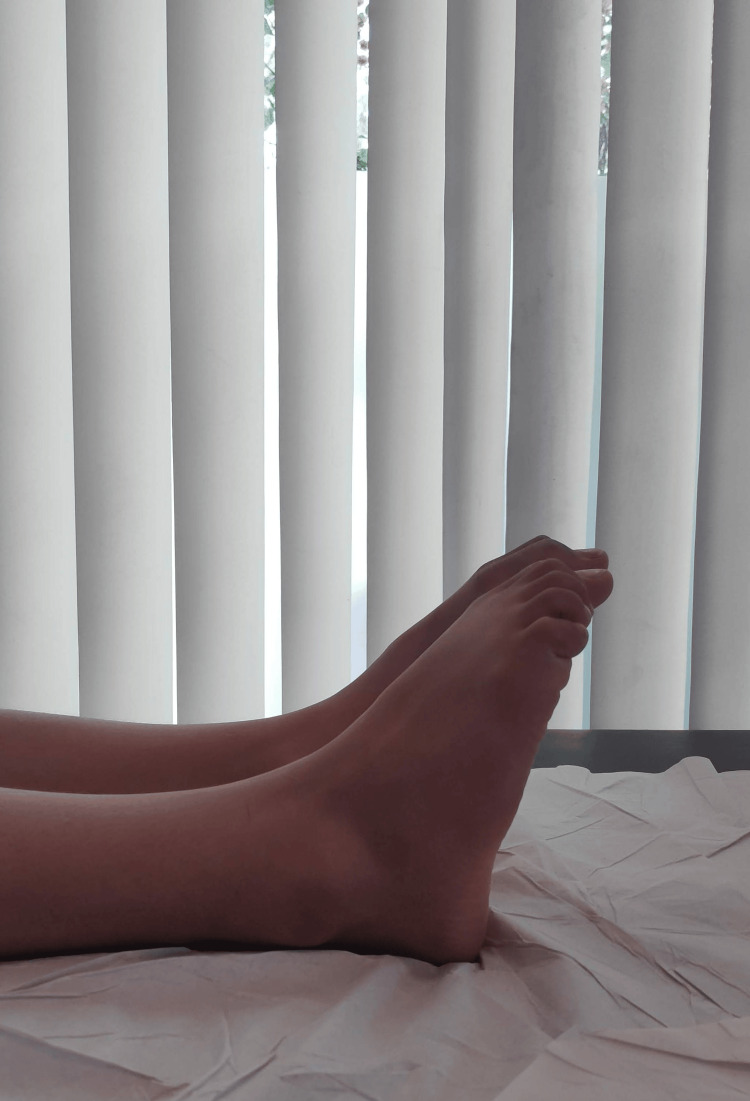
Preoperative photo of ankle showing 15 degrees of plantarflexion

On examination, there was diffuse hard swelling over the anterior ankle joint line with prominent lateral malleolus and restricted range of motion with no dorsiflexion beyond neutral, as shown in Figure 1, and plantar flexion up to 15 degrees, as shown in Figure 2. 

**Figure 3:**
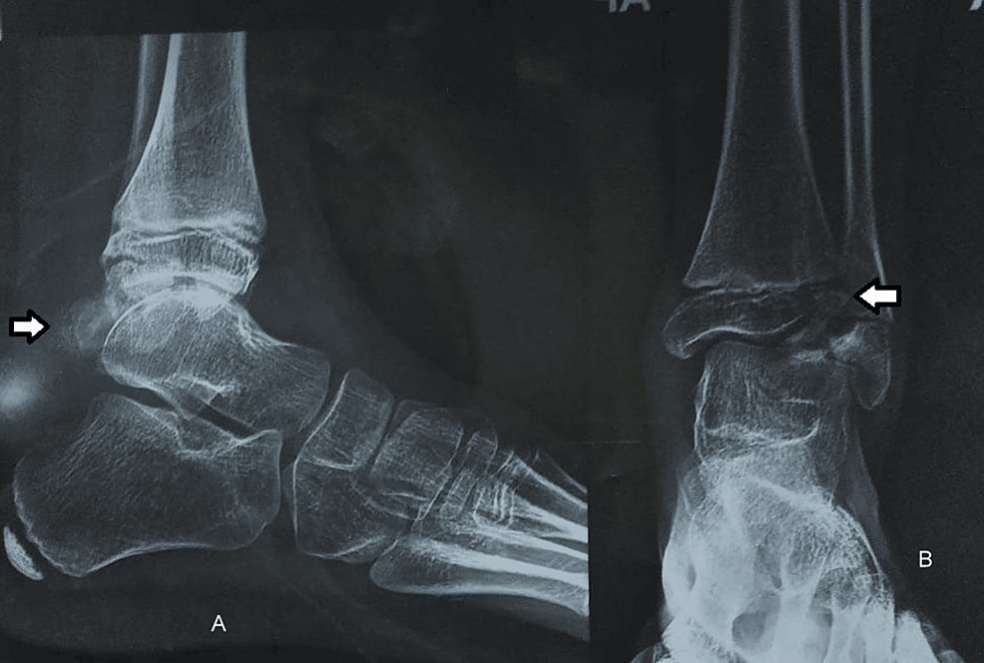
Preoperative lateral (A) and AP (B) radiographs of the ankle show bony outgrowths from the talus, which are directed posteriorly and laterally

Radiological investigation provided the following picture. A lateral view (Figure 3A) and AP view (Figure 3B) X-ray of the preoperative ankle demonstrates a bony outgrowth from the talus directed posteriorly and laterally.

**Figure 4:**
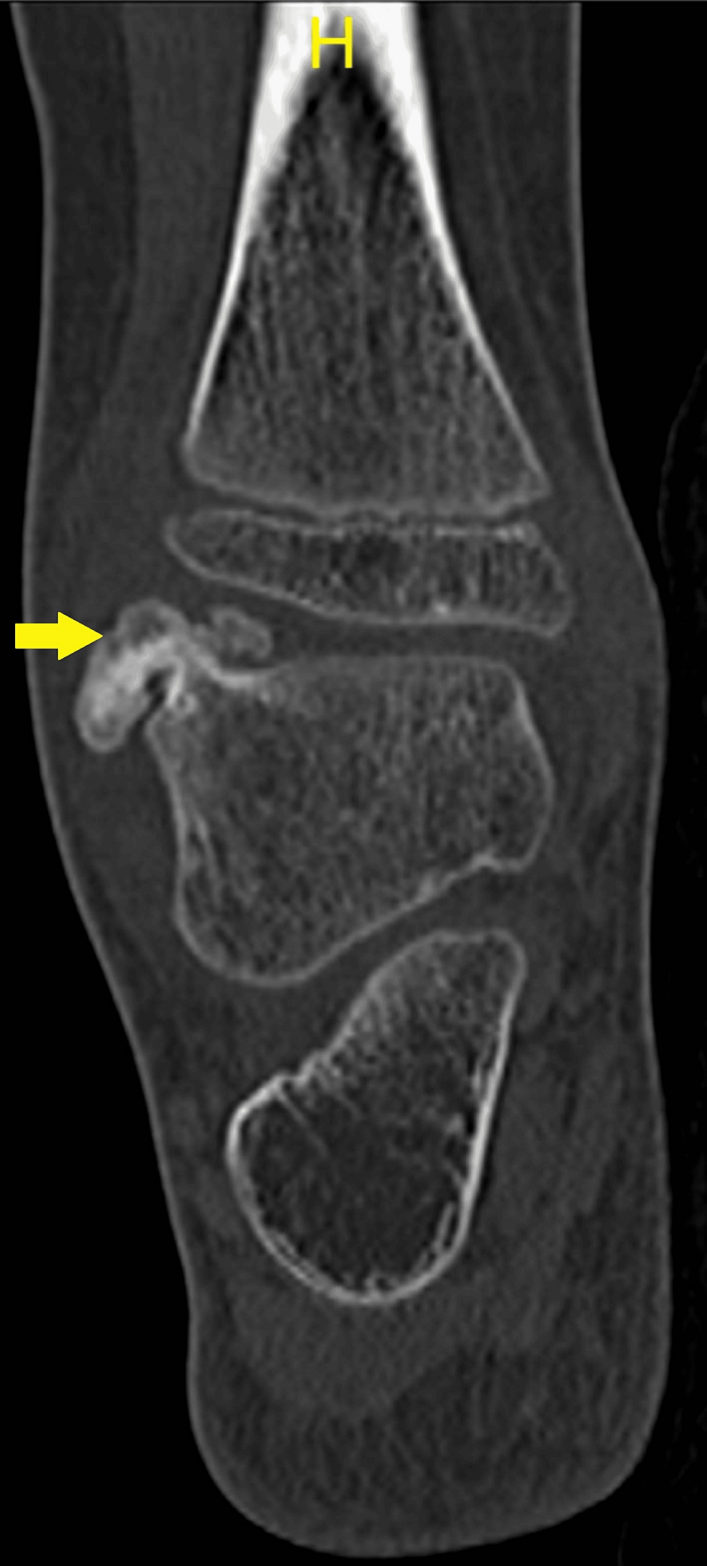
CT coronal view: arrows demonstrate the osteochondromas from the posterior aspect of the talus and in the interosseous region

**Figure 5:**
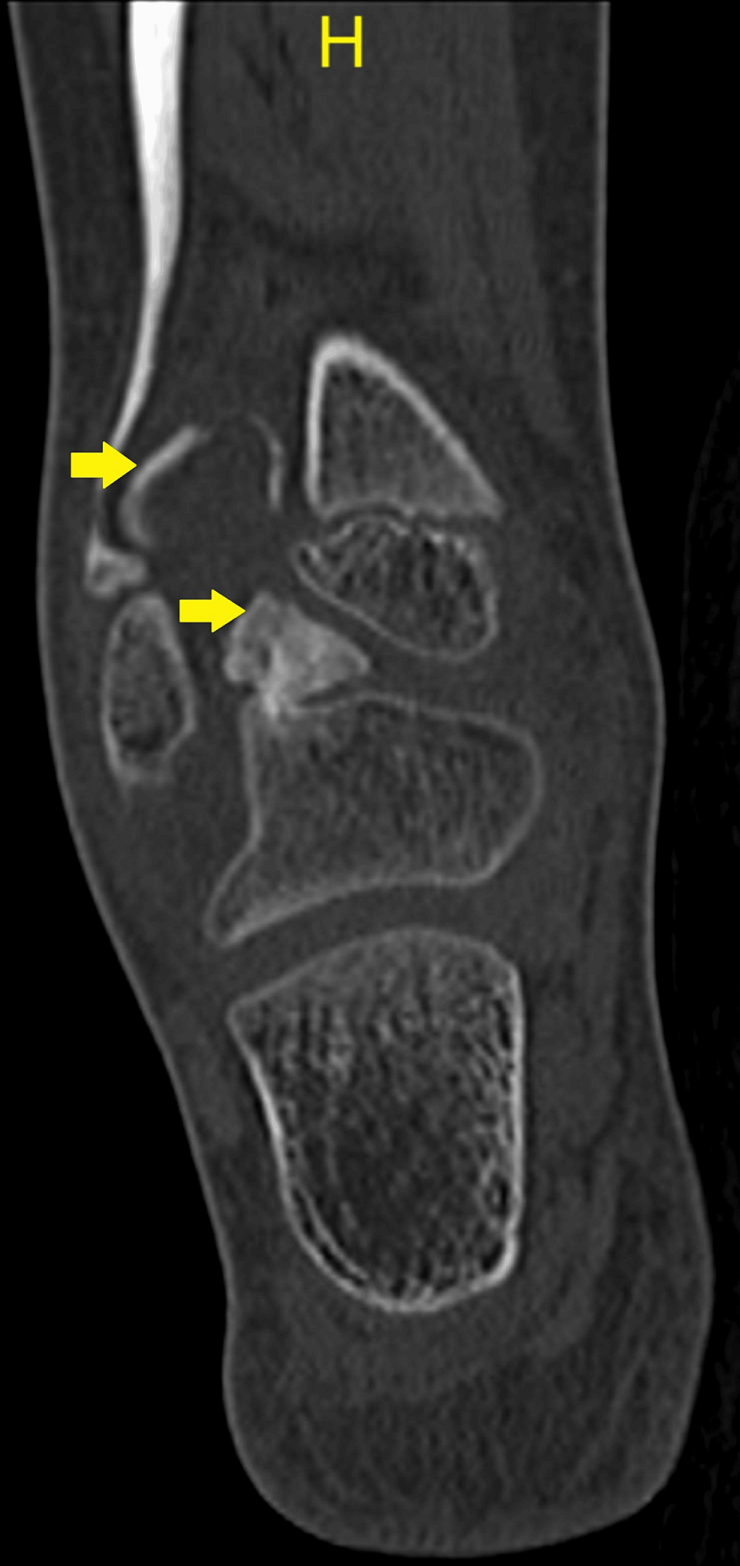
CT coronal image: arrows demonstrate the osteochondromas from the posterior aspect of the talus and in the interosseous region

**Figure 6:**
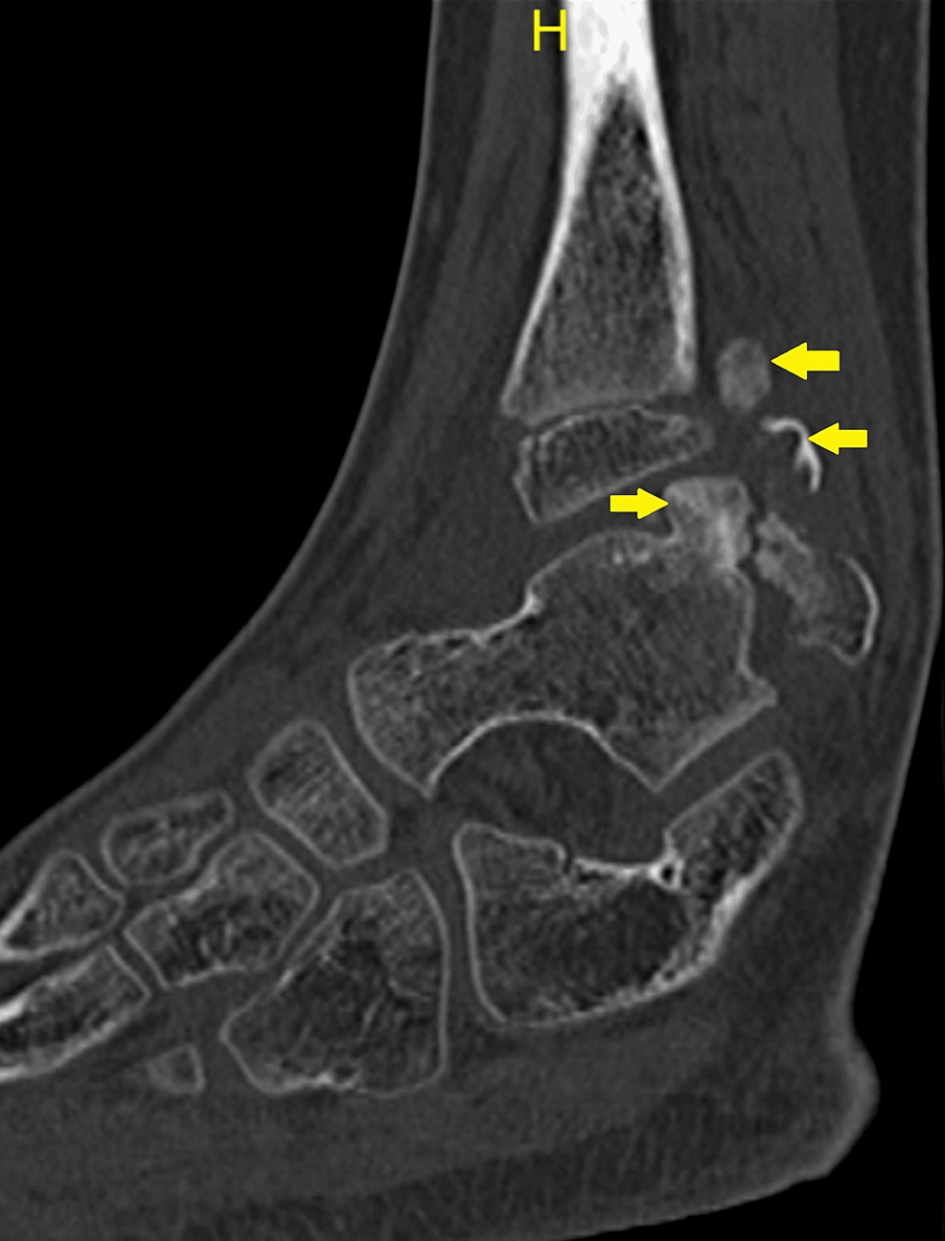
Sagittal image demonstrating the osteochondromas (arrows) from the posterior aspect of the talus and in the interosseous region

A CT scan revealed a bony tumor arising from the talar dome and extending into syndesmosis, as seen in Figure 4 and Figure 5, which are the coronal images, and Figure 6, which is the sagittal view.

**Figure 7:**
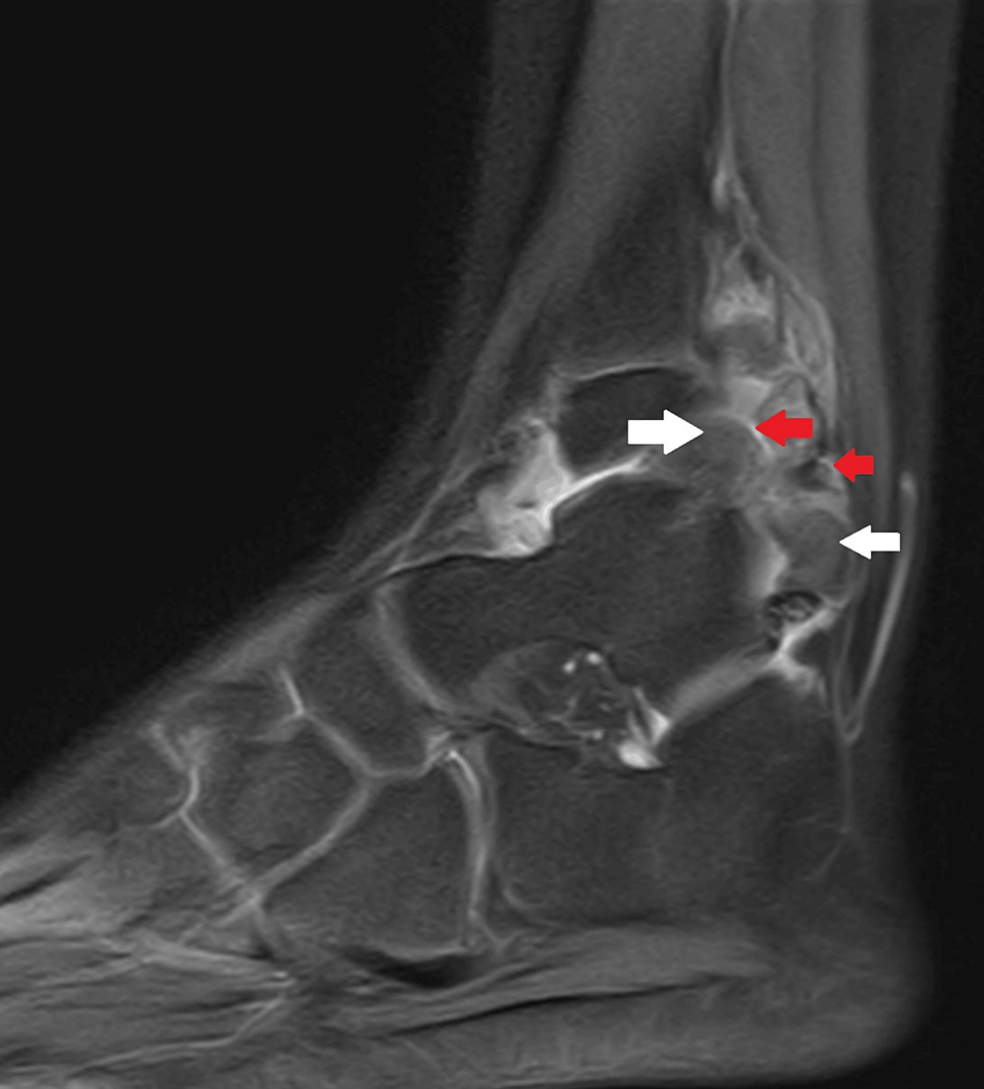
MRI proton density fat-suppressed sagittal images demonstrate the osteochondromas (white arrows) in the interosseous region. The thin cartilaginous cap of the osteochondromas can be seen as a hyperintense signal (red arrows)

**Figure 8:**
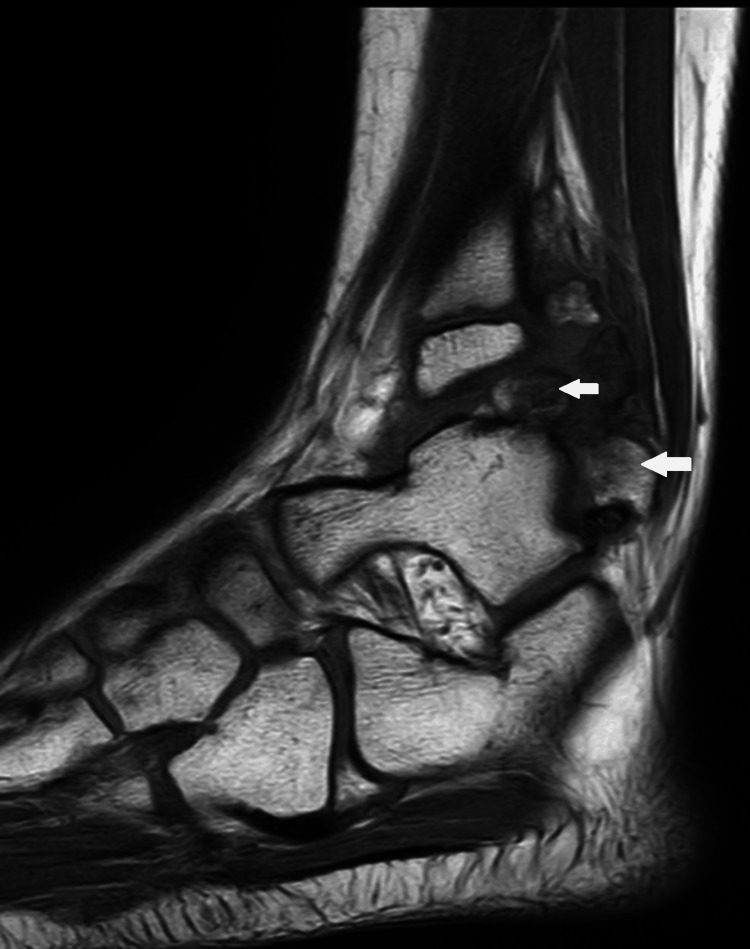
T1 sagittal images demonstrate the osteochondroma (white arrows)

MRI revealed the complete extent of the tumor with the cartilage cap and surrounding soft tissue compression, as seen in Figure 7 and Figure 8. The posterior inferior tibiofibular ligament (PITFL) was found torn due to the pressure from the extensive tumor. 

Operative technique 

Under general anesthesia, the patient was positioned in a lateral position. The tumor was dissected in both the anterolateral and posterolateral approaches. This approach was chosen based on preoperative radiological imaging. The extent and direction of the tumor were to be best approached by dual incisions, as mentioned above. Soft tissue dissection was done in layers to approach the bony lesion, taking care to avoid damage to the sural nerve in the posterolateral approach and the intermediate branch of the superficial peroneal nerve in the anterolateral approach. 

**Figure 9:**
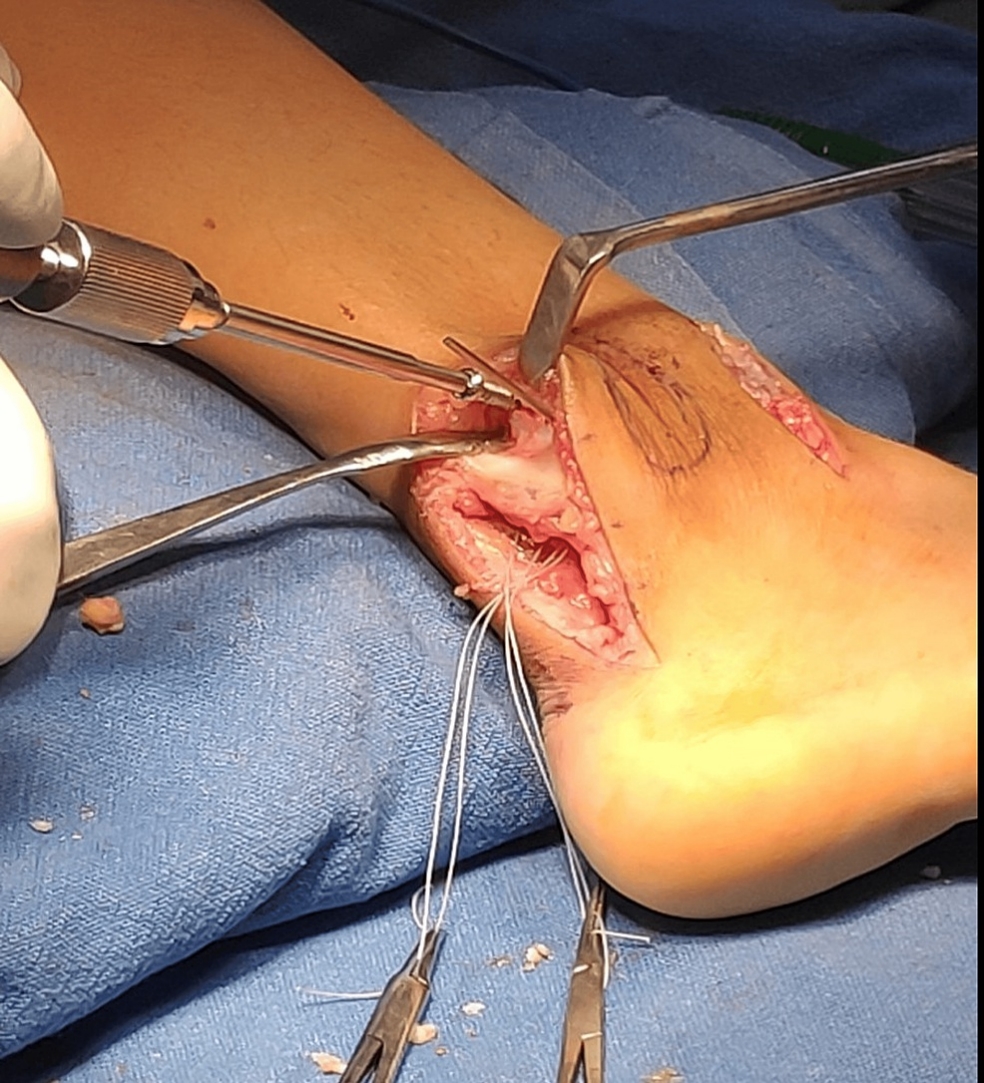
Intraoperative picture showing syndesmotic fixation and posterior inferior tibiofibular ligament (PITFL) repair

**Figure 10:**
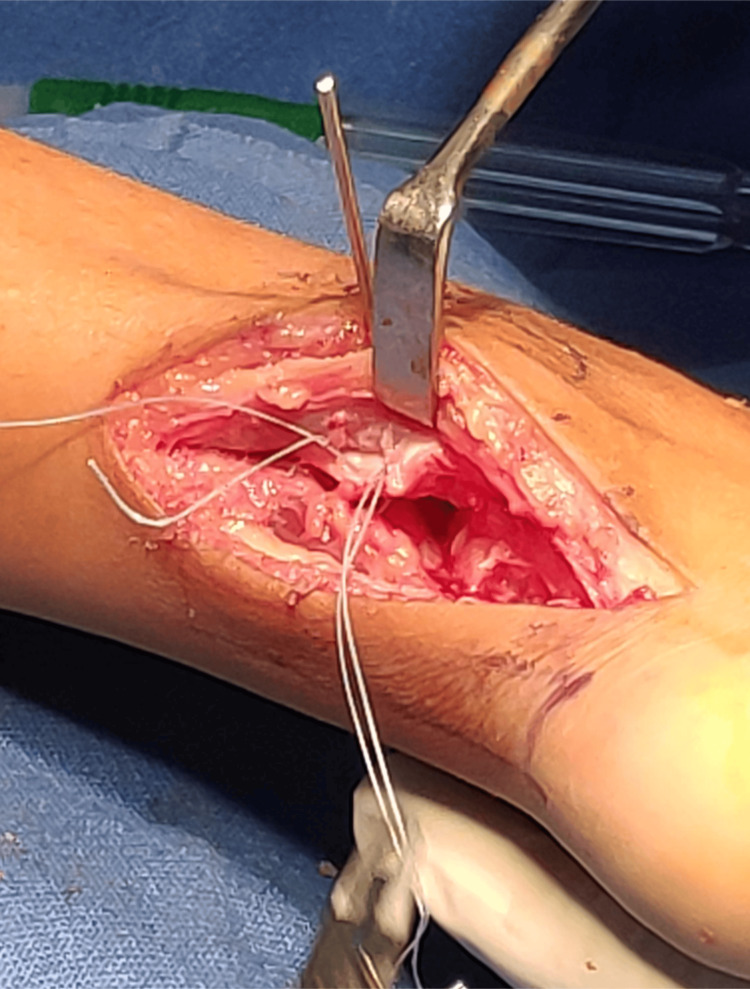
Intraoperative picture showing syndesmotic fixation and posterior inferior tibiofibular ligament (PITFL) repair

**Figure 11:**
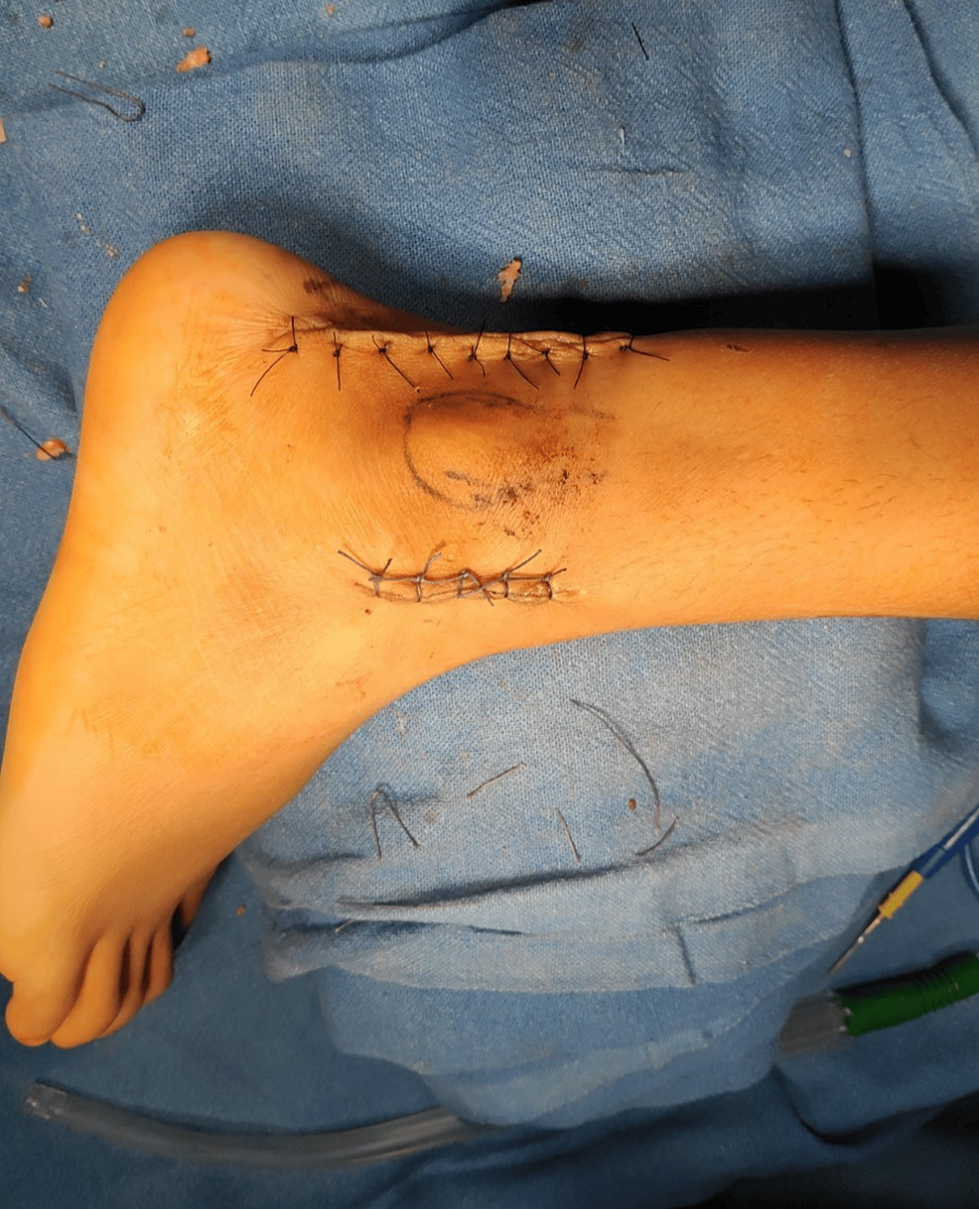
Wound after closure

Complete excision of two tumors of size 13x 11 mm and 11x 6mm was done, and completeness was confirmed under C-arm imaging. The excised tissue was sent for histopathological evaluation. After the excision of the tumor, there was a gap between the tibia and the fibula on direct visualization. When the ankle was checked for dorsiflexion and external rotation, there was further opening of the syndesmosis under intra-operative fluoroscopy in anteroposterior view. Hence, it was diagnosed intraoperatively that the syndesmosis was unstable. A decision was made to stabilize the syndesmosis with a 3.5 mm cortical screw, as seen in Figure 9. The posterior inferior tibiofibular ligament was seen to be ruptured and was repaired anatomically with fiber wire no 2 for added syndesmotic stability (Figure 10). Skin and subcutaneous tissue were closed in layers (Figure 11). 

The postoperative period was relatively uneventful; edge necrosis of the posterolateral wound occurred, which completely healed by secondary intention in two weeks. Histopathology was reported as cartilage-capped mature lamellar bone with enchondral ossification, which is consistent with osteochondroma. No evidence of malignancy seen". He was started on ankle range of motion exercises after two days of surgery and was not allowed to bear weight. When not exercising, he was immobilized with a removable splint. At three weeks, he was allowed to partially bear weight as tolerated. When reviewed at six weeks, he had progressed to full weight bearing and was not experiencing any pain.

**Figure 12:**
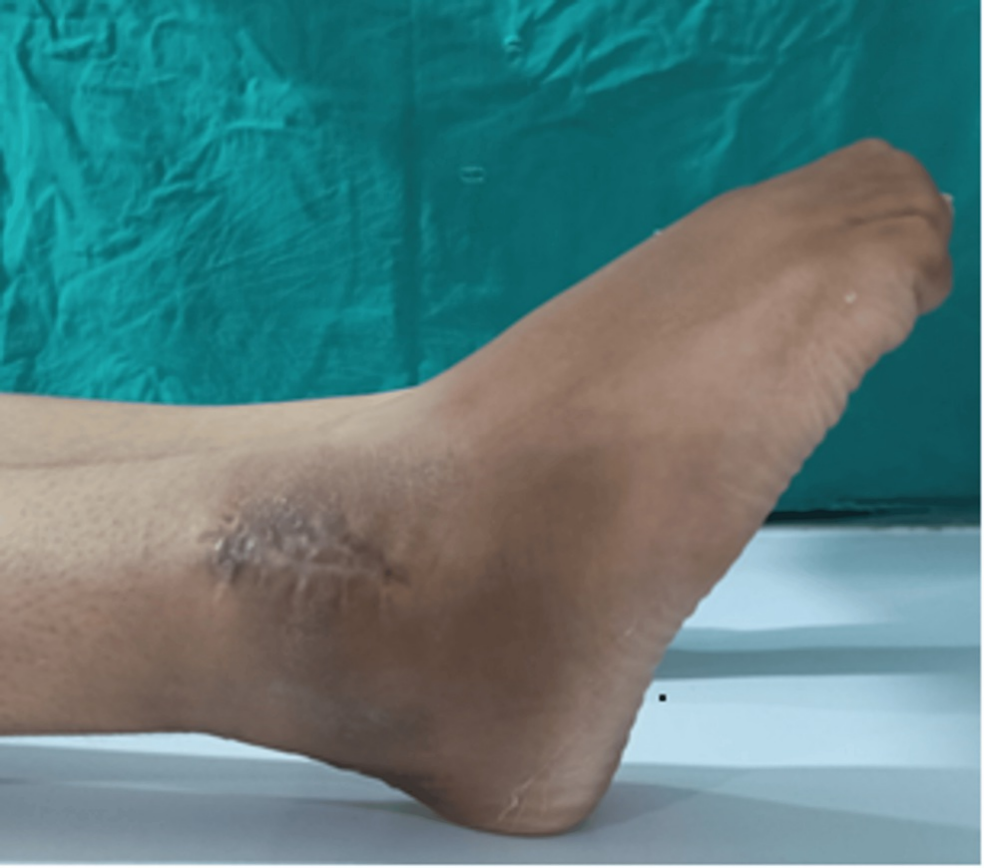
At six months follow-up, plantarflexion was seen improved to 40 degrees

**Figure 13:**
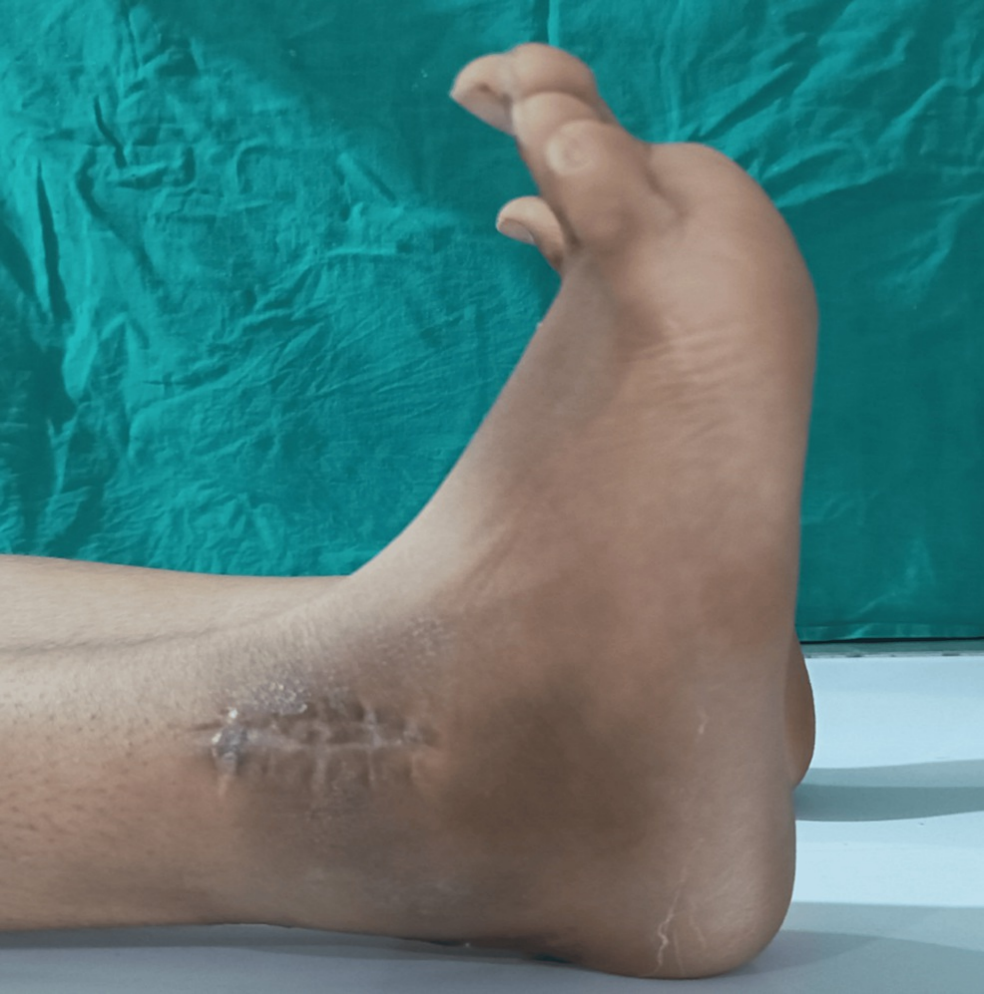
Ankle range of motion seen improved by 10-degree dorsiflexion

**Figure 14:**
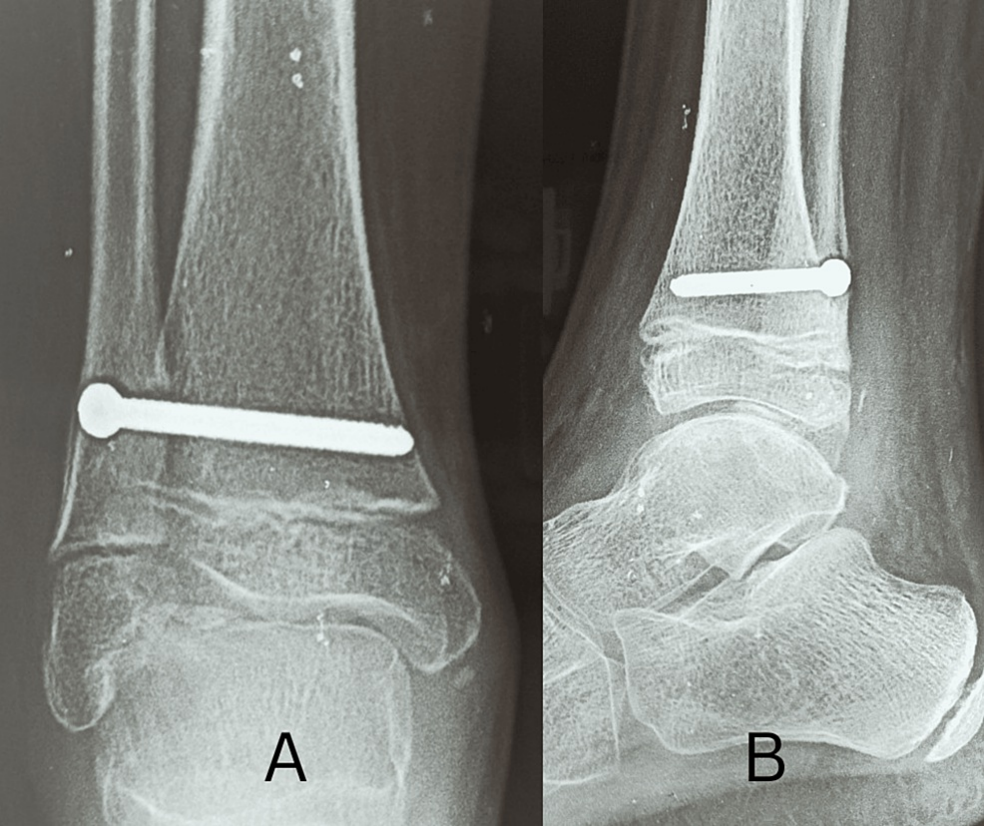
Six months follow-up AP (A) and lateral (B) radiographs showing good reconstruction of mortise

At six months follow-up, the range of motion had improved significantly to 30 degrees of plantar flexion (Figure 12), 5 degrees of dorsiflexion (Figure 13), and full range of inversion and eversion. An X-ray was taken of the ankle at six months (Figure 14), which showed good tibiofibular overlap in the AP without any recurrence of the tumor. 

 

## Discussion

The prevalence of osteochondromas remains uncertain due to many asymptomatic cases. Incidence varies by type, with solitary osteochondroma being about six times more common than hereditary multiple exostosis (HME). Most cases are seen within the first four decades, with 75% appearing before age 20, more often in males [3].​ 

Nearly half of osteochondromas are near the knee. Long bones are primarily affected, with distal femur (30%), proximal tibia (15-20%), and humerus (10-20%) being common sites. Osteochondromas are typically located in the metaphysis, away from joints. Flat bones like sternum, scapula, ribs, and hips are rarely affected (less than 5%) ​[4,5]. 

Talar osteochondromas are extremely rare, and only a few cases are reported in literature. It was first reported by Fuselier et al. in 1984 ​[6]. Chiorios et al. in 1987 reported an osteochondroma arising from the posterior aspect of the talus ​[7].​ Erler et al. reported a case of osteochondroma arising from the dorsum of the talus [8]. Our case was found to arise from the dorsal and posterior aspects of the talus. A study by Ozdemir et al. revealed that out of 1786 cases of osteochondromas, only 196 were found in the foot and ankle. These cases mainly originated from the metatarsus and phalanges [9].​ The Scottish Bone Tumor Registry conducted a review over 56 years and identified 23 cases of talar bone tumors. Among these cases, only two were confirmed to be osteochondromas ​[10]. There is no report describing an extension into syndesmosis. 

Osteochondromas typically occur during growth in individuals, arising from cartilaginous overgrowth near the growth plates. They can also develop as a result of hematopoietic stem cell transplantation or injury from surgery or radiation. The disease has a known genetic cause, with the tumor suppressor genes EXT1 and EXT2 implicated in its development [11].​ 

Solitary osteochondroma has traditionally been believed to carry a minimal risk of undergoing malignant changes. Changes in symptoms, particularly the emergence of pain in a previously pain-free lesion and an acceleration in its growth rate, should always be viewed as an alert for the potential of malignant transformation. Previous studies on osteochondromas have shown that the thickness of the cartilage cap serves as a valuable marker for assessing the likelihood of malignancy. Suspicion of malignancy arises if the thickness of the cartilage cap exceeds 2 cm in adults and 3 cm in children. Signs of secondary changes of osteochondroma should also encompass the consideration of erosion or damage to adjacent bones ​[12]. In our case, the cartilage thickness was 1.2 cm, and although extension into the syndesmotic joint was present with deformation of the distal fibula, there was no adjacent bony erosion. Diagnosis was confirmed as benign by histopathological evaluation. 

Suranigi et al., in their case report, described a dual approach of posteromedial and posterolateral incision to completely resect an extensive talar osteochondroma presenting as tarsal tunnel syndrome [13]. Erler et al., in their case report, describe using an anteromedial approach to excise a 1.5 cm talar osteochondroma arising from the anterior superior aspect of the talar body, which did not cause any soft tissue involvement​ [8]. Joshi et al. described a medial talar body osteochondroma causing pressure symptoms and weakness of the tibialis posterior muscle, which was excised using a medial approach [14]. Our case is the only reported case in literature where there is a syndesmotic extension with disruption of the interosseous membrane and PITFL ligament. We excised the tumor using an anterolateral and posterolateral approach and stabilized the syndesmosis with a cortical screw and PITFL anatomical repair. 

Postoperatively, he was started on ankle range of motion exercises after two days of surgery and was not allowed to bear weight. When not exercising, he was immobilized with a removable splint. Follow-up was done at two weeks for suture removal, two weeks for starting partial weight-bearing mobilization along with X-rays, and at six weeks for full weight-bearing and X-rays. X-rays were taken to look for overlap between the distal tibia and fibula, suggesting good syndesmotic stability. Range of motion was assessed, and progress was noted and documented at six weeks and six months. X-rays were compared between the immediate post-op ones and six-month follow-up ones to look for similarities between the overlap of distal tibia and fibula in AP views. Clinical examination for ankle stability and improvement of range of motion and pain was also done at six weeks and six months.

## Conclusions

A talar osteochondroma is a rare entity and presents as a syndesmotic extension causing disruption of the interosseous membrane; PITFL and intraoperatively detecting a syndesmotic instability is a first in literature as per our knowledge. A thorough preoperative radiological assessment was crucial in planning the approach and keeping instrumentation ready for syndesmotic stabilization after its excision. The patient was treated with complete excision and stabilization of syndesmosis with a cortical screw and anatomical PITFL ligament repair. Baring a delay in wound healing, a good functional outcome at six months in terms of gait and ankle range of motion has shown that the above preoperative approach for planning and treatment protocol can be taken as a reference for similar presentations in the future.
